# Genome Sequence of a Gyrovirus Associated with Ashy Storm-Petrel

**DOI:** 10.1128/MRA.00958-18

**Published:** 2018-09-20

**Authors:** Kara Waits, Russell W. Bradley, Pete Warzybok, Simona Kraberger, Rafaela S. Fontenele, Arvind Varsani

**Affiliations:** a The Biodesign Center for Fundamental and Applied Microbiomics, Center for Evolution and Medicine, School of Life Sciences, Arizona State University, Tempe, Arizona, USA; b Point Blue Conservation Science, Petaluma, California, USA; c Structural Biology Research Unit, Department of Clinical Laboratory Sciences, University of Cape Town, Cape Town, South Africa; Loyola University Chicago

## Abstract

Ashy storm-petrels (order Procellariiformes) are seabirds that are found along the coast of California to Baja Mexico. A novel gyrovirus was identified from a cloacal swab of an ashy storm-petrel, which is the second gyrovirus to be identified in sea birds, the first being found in the related northern fulmar.

## ANNOUNCEMENT

The ashy storm-petrel (*Oceanodroma homochroa*) is a seabird of conservation concern that is endemic to the California Current between western Baja California, Mexico, and northern California (1), with breeding populations concentrated at the South Farallon and Channel Islands (2, 3). The South Farallon Islands, 42 km west of San Francisco in the United States and part of the Farallon Islands National Wildlife Refuge, represent the largest colony, with ∼40% to 50% of the world population (3) and high visitation during the spring and summer (1, 2).

No viruses have been identified in ashy storm-petrels to date. As part of a viral discovery project, 40 individual cloacal swabs were collected in 2012 from adult ashy storm-petrels inhabiting the Farallon Islands and stored in RNAlater and guanidinium-isothiocyanate buffer. A 100-µl aliquot from each sample was used for viral DNA extraction as previously described (4, 5), and circular molecules were enriched by rolling-circle amplification using TempliPhi 100 amplification (GE Healthcare, USA). The resulting DNA was used to construct a 2 × 150-bp library using the Illumina TruSeq Nano DNA library prep kit and sequenced on an Illumina HiSeq 4000 platform at Macrogen, Inc. (South Korea). The raw reads (13,328,366 paired-end reads) were trimmed using Trimmomatic (6) and then *de novo* assembled using ABySS 2.0 (7). In the resulting 227,835 contigs (*N*_50_, 5,545 nucleotides [nt]), which were predominately *Pseudomonas* spp., an 818-nucleotide contig (with 15× coverage) was identified as having similarities to gyrovirus sequences using BLASTx (8). Gyroviruses (family *Anelloviridae*, genus *Gyrovirus*) are small, circular, negative-sense, single-stranded DNA viruses that have GC-rich noncoding regions, high sequence variability, and conserved genome organization (9). Relatively few gyroviruses have been identified, and little is known about their impacts on host organisms. Chicken anemia virus has been shown to cause immunosuppression, anemia, and hemorrhaging in young chickens (10). Using metagenomic approaches, various novel gyroviruses have been identified from chickens, human feces (in Chile, China, France, Hong Kong, South Africa, and Tunisia), a northern fulmar (in spleen and uropygial gland tissue [United States]), and ferret feces (in Hungary); however, no direct disease correlations have been demonstrated (11–16).

**FIG 1:**
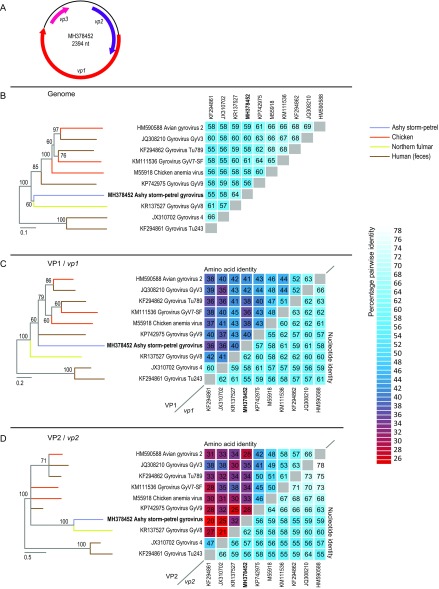
(A) Organization of the genome of ashy storm-petrel-associated gyrovirus VP1 (putative capsid protein; 1,368 nucleotides), VP2 (unknown function; 699 nucleotides), and VP3 (unknown function; 273 nucleotides). (B) Neighbor-joining phylogenetic tree of representative sequences (NCBI RefSeq) of gyroviruses with 1,000 bootstrap replicate branch support and pairwise identity matrix. (C) Maximum likelihood phylogenetic tree of the VP1 amino acid sequences and the pairwise identities of the VP1 protein and VP1 nucleotide sequences of representative gyroviruses. (D) Maximum likelihood phylogenetic tree of the VP2 amino acid sequences and the pairwise identities of the VP2 protein and VP2 nucleotide sequences of representative gyroviruses. The maximum likelihood phylogenetic trees were inferred using PHYML (17) with the WAG+G substitution model, determined as the optimal model using ProtTest (18), and the pairwise identities were inferred using SDT v1.2 (19).

Based on the gyrovirus-like *de novo*-assembled contigs, a set of back-to-back (for recovery of circular genomes) primers (5′-GTTACTTTCCAAGGTATTATTCTCATCCCC-3′, 5′-TCCGAGTGAGTTGTATGGTTTGGTAAC-3′) was designed and used to amplify the full genome of the gyrovirus. The cloned and Sanger-sequenced genome of ashy storm-petrel-associated gyrovirus (ASPaGyV) is 2,365 nucleotides in length, containing three large open reading frames (ORFs; VP1, VP2, VP3) (Fig. 1A). Representative genome sequences of gyroviruses were analyzed with that of ASPaGyV. The genome of ASPaGyV1 is most closely related to the gyrovirus GyV8 (GenBank accession number KR137527) from a northern fulmar, sharing 64% genome-wide pairwise identity (Fig. 1B). The VP1 of ASPaGyV shares 36% to 42% amino acid and 57% to 61% nucleotide identities with the VP1s of other gyroviruses (Fig. 1C). The VP2 shares 20% to 38% amino acid and 56% to 59% nucleotide identities with the VP2s of other gyroviruses (Fig. 1D). No homologues for the VP3 of ASPaGyV were identified. The pathology in ashy storm-petrels associated with ASPaGyV is unknown, and further work is necessary to determine the incidence rate and diversity of these viruses in these wild birds.

### Data availability.

The complete genome sequence of the ashy storm-petrel-associated gyrovirus isolate was deposited at GenBank under the accession number MH378452.
